# Erratum: Yuan, W., et al. Genome-Wide Identification of Banana *Csl* Gene Family and Their Different Responses to Low Temperature between Chilling-Sensitive and Tolerant Cultivars. *Plants* 2021, *10*, 122

**DOI:** 10.3390/plants10040654

**Published:** 2021-03-30

**Authors:** Weina Yuan, Jing Liu, Tomáš Takáč, Houbin Chen, Xiaoquan Li, Jian Meng, Yehuan Tan, Tong Ning, Zhenting He, Ganjun Yi, Chunxiang Xu

**Affiliations:** 1 Department of Pomology, College of Horticulture, South China Agricultural University, Guangzhou 510642, China; weina_yuan@stu.scau.edu.cn (W.Y.); liujing@stu.scau.edu.cn (J.L.); hbchen@scau.edu.cn (H.C.); mengjian@stu.scau.edu.cn (J.M.); yehuantan@stu.scau.edu.cn (Y.T.); ningtong@stu.scau.edu.cn (T.N.); hezhenting@stu.scau.edu.cn (Z.H.); 2 Centre of the Region Haná for Biotechnological and Agricultural Research, Department of Cell Biology, Faculty of Science, Palacký University Olomouc, 783 75 Olomouc, Czech Republic; tomas.takac@upol.cz; 3 Institute of Biotechnology, Guangxi Academy of Agricultural Sciences, Nanning 530007, China; lixiaoquan@gxaas.net; 4 Institute of Fruit Tree Research, Guangdong Academy of Agricultural Sciences, Guangzhou 510640, China

The authors wish to make the following corrections to this paper [1]:

The affiliation of Tomáš Takáč should have been:

Centre of the Region Haná for Biotechnological and Agricultural Research, Department of Cell Biology, Faculty of Science, Palacký University Olomouc, 783 75 Olomouc, Czech Republic; tomas.takac@upol.cz

The authors apologize for any inconvenience that may have been caused.

## References

[1] Yuan W., Liu J., Takáč T., Chen H., Li X., Meng J., Tan Y., Ning T., He Z., Yi G. (2021). Genome-Wide Identification of Banana Csl Gene Family and Their Different Responses to Low Temperature between Chilling-Sensitive and Tolerant Cultivars. Plants.

